# Editorial: Insights in Cushing’s syndrome and disease, volume II

**DOI:** 10.3389/fendo.2024.1500755

**Published:** 2024-10-31

**Authors:** Fabienne Langlois

**Affiliations:** Department of Medicine, Division of Endocrinology, Centre Intégré Universitaire de Santé et de Services Sociaux de l'Estrie, Centre Hospitalier Universitaire de Sherbrooke, Sherbrooke, QC, Canada

**Keywords:** Cushing syndrome, Cushing disease (CD), cortisol, hypercortisolism, adrenal Cushing syndrome, ectopic Cushing syndrome

## Introduction

Endogenous Cushing’s syndrome results in significant multisystemic morbidity and elevated mortality if left untreated (1). The manifestations of this condition impact multiple organs and systems, and the clinical presentation of the disease varies widely among individuals. A high index of clinical suspicion is essential for accurate diagnosis, which necessitates multiple sequential tests, each requiring careful interpretation (2). The complexity of diagnosing and managing this condition demands individualized approaches and presents ongoing challenges for endocrinologists (3).

## Aims and objectives

This Research Topic encompasses original studies addressing critical clinical questions aimed at improving the diagnosis and treatment of patients with Cushing’s syndrome. The objective is to gather novel insights into the disease to assist clinicians in patient management and to inform research initiatives.

## Overview of the contributions

The study by Pekul et al. emphasized the relevance of USP and TP53 mutations in pituitary tumors for prognostic guidance by comparing corticotroph tumors causing Cushing’s disease (CD) with their silent counterparts.

A meta-analysis by Giampetro et al. evaluated the use of desmopressin for diagnostic purposes, a review that is particularly relevant given the limited availability of CRH in many countries. The article discussed the strengths and limitations of interpreting the 10mcg desmopressin test in differentiating between ACTH-dependent Cushing’s syndrome and non-neoplastic hypercortisolism. Additionally, a retrospective cohort study presented by Skrebsky De Almeida et al. investigated the role of desmopressin during Bilateral Inferior Petrosal Sinus Sampling (BIPSS), presenting sensitivity and specificity values of the central-to-peripheral ratio before and after stimulation.

Wright et al. provided a review of emerging diagnostic methods, including advanced functional imaging and measurement of hair cortisol and cortisone, addressing the limitations of current tests while highlighting the advantages of novel approaches with the potential to transform the investigation of the condition.

The impact of hypercortisolism on the gut microbiota was explored in a case-control study by Valassi et al. that offered insights into the ongoing cardiometabolic consequences of Cushing’s syndrome.

Jurek et al. conducted a prospective study of the hemodynamic profile of newly diagnosed CD patients, analyzing results by sex.

Feelders et al. shared the results of the extension of a multicenter Phase II study in which patients were initially treated with pasireotide which was subsequently combined with cabergoline if cortisol levels remained elevated. The study demonstrated that this combination of pituitary-targeted therapies can yield sustained long-term efficacy in selected patients.

Zhang and Ioachimescu discussed the post-surgical recovery phase in a mini review, highlighting the need for clinicians to anticipate the glucocorticoid withdrawal syndrome and adequately prepare their patients for this challenging phase, during which symptoms may initially worsen before improving. This review presents novel data to enhance understanding of the condition and suggests management strategies.

## Call to action

This Research Topic highlights several key areas of ongoing research aimed at advancing our understanding of Cushing’s syndrome. It underscores the importance of collaborative efforts to improve the health and quality of life of patients affected by this condition.
